# Cervical Ribs

**Published:** 1910-04-30

**Authors:** 


### Cervical Ribs.

The curious congenital abnormality in which an extra rib (or a pair of them) springs from the seventh cervical vertebra is always a source of interest when encountered in the dissecting room by the student learning anatomy. The event is sufficiently unusual in man, and sufficiently difficult of explanation, to make it worth some keen comparative biologist's while to investigate its occurrence and frequency among the members of the order *Primates*. But to the clinician cervical ribs have an interest of an entirely different kind, for to him speculations as to the phylogenesis of an extra pair of ribs are of less importance than the diagnosis, symptoms, and treatment of the condition.

That this condition is rarely diagnosed will be conceded without demur, but that it is actually so extremely rare is not quite so certain. It is, admittedly, very uncommon, but since the widespread adoption of $x$-rays as an aid in diagnosis, the condition has been recognised with distinctly greater frequency than formerly. The symptoms are frequently so obscure that the assistance of a radiologist may easily fail to suggest itself to the mind of the clinician; probably in a fair number of cases the neuritic and other symptoms are too mild seriously to impair the comfort and usefulness of the patients in their work, and the latter consequently do not make sufficient complaint to lead their doctors to think of cervical ribs.

There are few disorders in which accurate diagnosis depends so completely upon an $x$-ray photograph, and fewer still in which the evidence so obtained is more conclusive or less ambiguous.

Two papers recently published are valuable reminders of the existence and the latency of this peculiar clinical entity. One is from the pen of Professor Osler*; the other by Mr. Seymour Barling.† The latter surgeon describes the case of a boy of seven years of age, who presented himself at the Birmingham Children's Hospital for a tumour above the left clavicle, which had been discovered by a school medical officer in his routine examination. On the right side an undue fulness was also evident, and the outline of a rib could be indistinctly palpated. On the left side, where the larger tumour was, the diagnosis of a cervical rib was made more easily, as the bone was traceable from the spine round to an angle where it dipped sharply down towards the first thoracic rib; it was this angle which caused the prominence in the posterior triangle of the neck. No pressure symptoms of any kind, nervous or vascular, existed. The author notes, however, that, although surgical interference seems at present to be unnecessary, continuation of growth from the epiphysis may easily render removal imperative at some future date. In the literature he finds the earliest age at which symptoms have supervened to be sixteen, and the oldest sixty; but the usual onset of trouble from a cervical rib is between twenty-five and thirty-five.

Professor Osler's paper is based on two cases which he observed during 1909; he reports one of them fully because he thinks it throws light on certain obscure cases seen by him many years ago, in which at the time the possibility of cervical ribs did not occur to him. The patient was a woman of thirty-one, who for ten or twelve years had noticed pulsation above the clavicles, especially the left. Her physicians had discussed the possibility of aneurysm. For some months she had suffered from numbness, "pins and needles," swelling, and inability to use the arm. These symptoms came on only after free use of the arm, not during rest; the use of the fingers did not provoke them. Sensation, movement, and all the functions of the limb were perfect. On palpation no definite tumour could be felt, yet arterial pulsation was very marked. On deep pressure, resistance suggestive of a cervical rib was found, a suspicion confirmed by $x$-ray examination. The author recalls two similar cases—one in Philadelphia and one in Baltimore—in which the symptoms of pain after use, flushing, tingling, and swelling of the arm were even more prominent than in the recent case; in both these cases the patients were unable to continue their occupation on account of the condition. At the time he did not suspect cervical ribs, and apparently the patients went unrelieved; but he is now convinced that this was the real cause of their troubles. To fail to diagnose one of these cases need scarcely be a matter of serious reproach to anyone when Dr. Osler admits having done so twice himself; but the peculiar value of his paper is that, having frankly admitted so much, he explains also the characteristic signs and symptoms for the guidance of others. His description is therefore worth bearing in mind whenever an unusually puzzling case of supraclavicular pulsation with brachial vascular and neuritic symptoms is brought to one for treatment.

The exact method of surgical treatment for the removal of a cervical rib must depend in some degree upon the anatomical peculiarities of the individual case. The text-books of operative surgery do not devote much attention to the subject; but on general principles it may be urged that in every case the symptoms should be very carefully considered, and that no more should be attempted than will, in the operator's judgment, suffice for the relief of the patient and the prevention of future trouble. To remove the entire rib would involve so much interference with the deep tissues of the neck as to be unnecessarily risky; provided that resection of a portion of it will relieve vessels and nerves from pressure, it would seem that more drastic measures are unsurgical.

* *American Journal of the Medical Sciences*, April 1910, p. 469.

† *British Journal of Children's Diseases*, March 1910, p. 108.

